# Report of the Obstetric Practice in the Philadelphia Dispensary for the Year 1840

**Published:** 1841-01-30

**Authors:** Joseph Warrington

**Affiliations:** Accoucheur


*Report of the Obstetric Practice in the Philadelphia Dispensary for the year* 1840. By Joseph Warrington, M. D., Accoucheur.

*One hundred and thirteen pregnant women* have been under care during the year. Of these 110 have been safely delivered at full term; 1 aborted at about five months, and 1 at about four months, in consequence of personal injury; 1 died undelivered, in an impracticable labour, resulting in rupture of the uterus.

*One hundred and thirteen children* have been delivered at term, there being three cases of twins—two with boys, and one with girls. The case of the male child, which died in utero, and the two cases of abortion, are not included in this enumeration.

Fifty-five of the children were boys, and fifty-eight were girls.

Four of these children were stillborn—the death of three appearing to be recent, while in one case there was no doubt but it had occurred some weeks previous to delivery.

Of the other children, one died in convulsions on the third day after its birth; one male child had its urethra blocked up by tuberculous deposit, which was relieved by the introduction of the catheter by Dr. Mütter, and finally cured by diuretics; one female child had occlusion of the meatus urinarius overcome by several introductions of the catheter by myself; one child had hare-lip successfully operated upon by Dr. Mütter; one had a large tumour on the scalp, respecting the propriety of removing which the surgical skill and experience of Dr. W. Poyntell Johnston, were solicited and obtained; one child had icterus, which was cured by minute doses of calomel and bicarbonate of soda. All the other children were doing well when they passed from under the care of the accoucheur.

Of ninety-nine cephalic presentations, fifty-two were in the first position of the vertex, fourteen in the second, one in the third, two in the fourth, and one in the fifth; this last was converted into a first position. Of the remaining twenty-nine the positions were not satisfactorily ascertained.

Of four pelvic presentations, two were in the second position, and one in the fourth position; the other was not satisfactorily diagnosticated.

In seventy-one cases of labour, the average duration was fourteen hours forty minutes,—the extremes being one and fifty-two hours.

The average amount of time occupied in the delivery of the placenta, in seventy-six cases, was forty-six minutes, the extremes being five minutes and eight hours.*

Ergot was administered in one case to terminate a labour, which had continued fifty-two hours. It was also given in a case of placenta prævia, to shorten the second stage of labour; also, in two cases of hæmorrhage, after extrusion of the child.

Delivery was accomplished by the use of the forceps in three cases, in which both mother and children did well. In a fourth case they were applied ineffectually, rupture of the uterus taking place, and the woman dying undelivered, apparently in consequence of the excessive size and firmness of the child's head.

There were two cases of profuse hæmorrhage, with retention of placenta, in which ergot was used for the purpose of exciting uterine contractions. Free friction and compression of the abdomen, and the removal of the placenta appeared to be the most efficient means of relief, the ergot seeming not to act in either case.

There was one case of adhesion of the edge of the placenta and a considerable portion of the membranes,—it was attended by very alarming hæmorrhage, which could not be arrested until the hand was introduced and the secundines removed.

There was one case of hæmorrhage during labour, from an implantation of the placenta upon the cervix uteri. The tampon was introduced and sustained by two assistants, who made constant pressure upon the perinæum with compresses, until the completion of the first stage of labour. Ergot and brandy were then freely administered, (the woman being prostrate from the loss of blood;) the membranes were ruptured artificially, and the hæmorrhage arrested by facilitating the descent of the breech (the original presentation) into the orifice of the uterus; the child was dead, apparently in consequence of the loss of blood through the placenta.

There were two cases of metritis; they recovered under the use of free bleeding, purging, fomentations, and diaphoretics.

There was one case of metro-peritonitis, which was cured by free and repeated abstractions of blood generally and locally, the use of blisters, the administration of calomel, Dover's powders, and the spirits of turpentine.

One patient (of strumous diathesis) became the subject of abscess in both mammæ, from which she recovered, one gland resuming its functions.

There was one case of abscess in the vagina or cervix uteri during pregnancy. It was relieved by mucilaginous injections per vaginam previous to delivery, and apparently cured by a recurrence to the same treatment some weeks after parturition.

Two cases of prolapsus uteri were observed to follow labour. In one case it was probably induced by the patient rising on the second day after delivery, to go into another room, to supply the wants of one of her children; the other patient had been subject to the affection previous to her late pregnancy.

One case of prolapsus vesicæ occurred without any assignable cause, shortly after a favourable labour of only eight hours' duration.

* It is proper to remark that the delay in the delivery of the placenta depended in most instances upon its being purposely left to the efforts of the uterus and the voluntary powers of the mother, care being taken not to interfere with the *natural process* of extrusion of placenta, with a view to determine the length of time it might require.

